# Transformation Versus Ascertainment Bias of a Suprasellar Lesion: A Histopathologic Conundrum of BRAF V600E Positive Papillary Craniopharyngioma Versus Rathke's Cleft Cyst with Squamous Metaplasia: A Systematic Review

**DOI:** 10.1055/a-2751-8340

**Published:** 2025-12-10

**Authors:** Hailey Mattheisen, Samon Tavakoli, Edward Kelly Mrachek, Stephanie Cheok, Nathan Zwagerman

**Affiliations:** 1 Department of Neurosurgery, Medical College of Wisconsin and Froedtert Hospital, Wauwatosa, Wisconsin, United States; 2 Department of Pathology, Medical College of Wisconsin and Froedtert Hospital, Wauwatosa, Wisconsin, United States

**Keywords:** CP, RCC, BRAF V600, histological transformation

## Abstract

**Background:**

Differentiating craniopharyngiomas (CPs) from Rathke's cleft cysts (RCCs) is challenging due to overlapping features. RCCs with squamous metaplasia (SM) may represent a transition to CPs, complicating diagnosis. This study presents a recurrent RCC later confirmed as papillary CP, prompting a systematic review to identify early diagnostic markers. The goal is to improve RCC and CP differentiation, preventing radical resection of true RCCs, and predicting recurrence or transformation to CPs.

**Methods:**

A systematic review was performed with adherence to Preferred Reporting Items for Systematic Reviews and Meta-Analyses guidelines. Using the PubMed/Medline databases, a search string was created with the keywords “RCC transformation or (RCC and CP) or (RCC to CP) or (RCC to CP) or (Rathke's and CP).” The initial search yielded 489 papers, narrowed by key data points including RCC recurrence with histologic CP confirmation.

**Results:**

The final review included five studies, which detailed cases of initial pathological diagnosis of RCC that were later diagnosed as a CP upon repeat surgery and tissue sampling. Histological examination of primary and secondary surgical resections revealed RCC recurrence with transformation to CPs (two adamantinomatous CPs, two papillary CPs, and one ciliated CP).

**Conclusion:**

RCCs and CPs share overlapping features, complicating preoperative diagnosis and treatment. RCC recurrence with subsequent CP is rare, as our review identified only five recorded cases. Definitive diagnosis requires pathology, though sampling bias poses challenges. Advanced imaging (contrast-enhanced 3D T2-FLAIR MRI) and biomarkers (BRAF V600E, beta-catenin, p53, Ki-67) show promise in improving diagnosis, predicting recurrence, and guiding treatment.

## Introduction

Accurate diagnosis of sellar lesions is paramount to developing appropriate management goals. The differential diagnosis is broad, and the most common tumors in this region include pituitary adenomas, meningiomas, Rathke's cleft cysts (RCC), and craniopharyngiomas (CP). 1 With advancements in imaging and other diagnostic techniques, most cases have a clear, favored diagnosis preoperatively. However, a subset of cases present with overlapping clinical, radiographic, and histopathologic features that make diagnosis challenging.

RCCs are benign, cystic lesions that arise from the remnants of the craniopharyngeal duct in Rathke's pouch. They are most often present in mid-adulthood with symptoms such as headaches, visual deficits, and hypopituitarism secondary to mass effect. 2 On imaging, RCCs are nonenhancing cysts of variable signal intensity secondary to proteinaceous contents of the cyst, but rarely associated with calcification. 3 4 While typically confined to the sella turcica, they may extend into the suprasellar space and, in some cases, into the sphenoid and cavernous sinuses. 5 6 CPs are benign, epithelial tumors that arise from the craniopharyngeal duct. 7 They demonstrate a bimodal age distribution, occurring in both children and older adults. 8 In the fifth edition of the WHO classification of central nervous system tumors, adamantinomatous craniopharyngioma (ACPs) and papillary craniopharyngiomas (PCPs) are classified as distinct entities given clear differences in demographics, histopathologic findings, and genetic profiles. 9 PCPs are much less common than ACPs and typically present in adulthood. ACPs are primarily suprasellar and may extend into surrounding locations. 10 On imaging, they tend to be cystic with calcifications. 4 10 11 PCPs tend to be primarily suprasellar with extension into the sella, third ventricle, and surrounding structures. On imaging, they enhance vividly and are typically solid with minimal cystic formation and calcification. 4 10 12

Because imaging findings alone may be insufficient to differentiate between RCCs and CPs, histological and immunohistochemical analysis remain critical for establishing a definitive diagnosis and guiding management. 13 14 Management paradigms for RCCs and CPs vary considerably. RCCs may be observed or surgically drained if symptomatic, whereas CPs usually require resection and, in select cases, adjuvant radiation. 15 16 Achieving accurate diagnosis is therefore central to optimizing patient outcomes. In this paper, we conduct a systematic review with the goal of uncovering a combination of factors that can ideally be used in the diagnostic schema for suspected RCCs and CPs.

## Methods

The systematic review was performed in accordance with the Preferred Reporting Items for Systematic Reviews and Meta-Analyses guidelines.

### Research Strategy and Selection Process

This review included all previously published literature comparing RCCs and CPs. Studies discussing the histological, clinical presentation, imaging studies, surgical approaches, treatment, or postoperative outcomes were evaluated. Reports describing the potential transformation of an RCC and CP were included. Eligible studies were published between 1951 and 2024 and written in the English language.

**Fig. 1:**
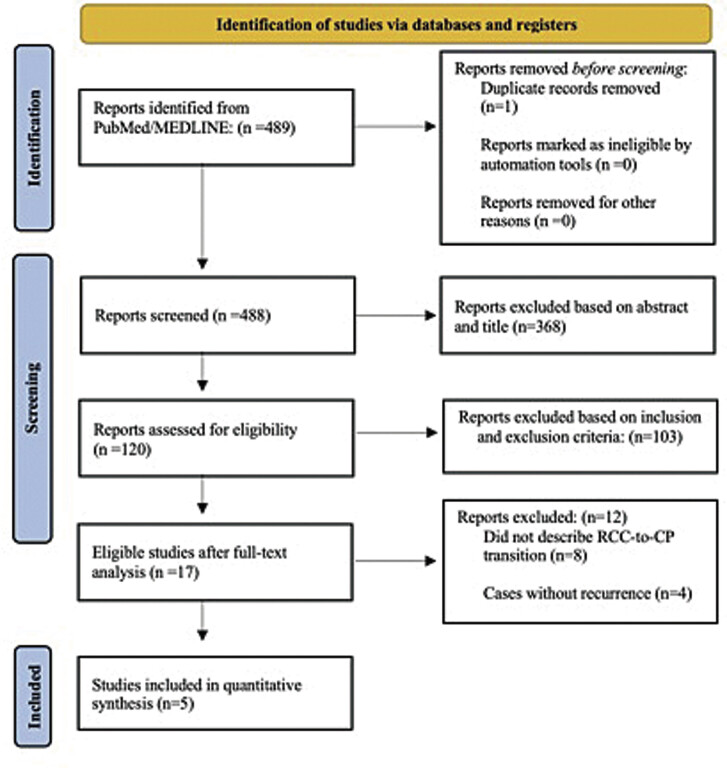
Prisma flow diagram illustrating the selection process of studies.

A comprehensive search was conducted using the PubMed/Medline databases with the following search string: “RCC transformation or (RCC and CP) or (RCC to CP) or (RCC to CP) or (Rathke's and CP).” The initial search identified 489 papers, and 1 surgical video published between 1951 and 2024. After preliminary review of titles and abstracts, 120 articles were retained for full-text evaluation ( Fig. 1 ).

### Inclusion Criteria

Two independent reviewers assessed the remaining studies for relevance, narrowing the selection to 17 articles that discussed RCC and CP recurrence. The review process was performed in three separate rounds to ensure accuracy and consensus. Studies were included if they described patients initially diagnosed with RCC who were later diagnosed with a histologically confirmed CP. The final five studies included for quantitative analysis met all inclusion criteria and contained sufficient detail to extract clinical, radiologic, histologic, and surgical data for both the initial and recurrent tumors ( Table 1 ).

**Table 1:** Summary table of the studies identified and included in our analysis from our systematic review.

Supporting study	Sex and age	Time to recurrence (mo)	Presence of SM after the first surgery	Diagnosis after the first surgery	Presence of SM after the second surgery	Diagnosis after the second surgery
Park et al 7	M, 41	34	No	RCC	No	ACP
Ogawa et al 41	M, 47	6	No	RCC	Yes	ACP
Okada et al 42	M, 61	3	Yes	RCC	Yes + B-catenin	CCP
Manjila et al 19	M, 46	1	No	RCC	NA	PCP
Sharma et al 43	F, 36	3	Yes	RCC	Yes + BRAF	PCP

### Exclusion Criteria

For our quantitative analysis, studies were excluded if they did not describe cases of true RCC-to-CP transformation. Specifically, reports were excluded if initial reports of RCCs recurred without subsequent histological confirmation of CP. Additionally, studies were excluded if RCC-to-CP transformation was speculative on preoperative radiologic findings alone or if insufficient clinical or pathological details were provided.

## Results

### Systematic Review Findings

Our systematic review identified five studies describing recurrent RCCs with transformation to CPs. Across these reports, there were four males and one female, with an average age of 46.2 years (SD = 9.36, range = 36–61). All patients initially presented with visual acuity loss or bitemporal hemianopsia. Endocrine dysfunction was reported in two cases, while pituitary function remained intact in the remaining three.

MRI findings were consistent with an initial diagnosis of RCC in all five cases, with notable suprasellar invasion in four patients. All patients underwent an extended endoscopic transsphenoidal approach for resection of their cystic lesions. Initial histology revealed squamous metaplasia (SM) in two patients, one of which was also tested for beta-catenin, which was negative., All five patients experienced recurrence with an average rate of 9.4 months (SD = 13.87, range = 1–34).

After undergoing additional surgery for cyst recurrence, all five patients initially diagnosed with having an RCC were subsequently diagnosed with having CPs (two ACPs, two PCPs, and one ciliated CP). This time, three of the patients showed histological evidence of SM, with two of them originally having SM present after initial surgical resection of the RCC. These findings are outlined in Table 1 .

### Institutional Case

**Fig. 2:**
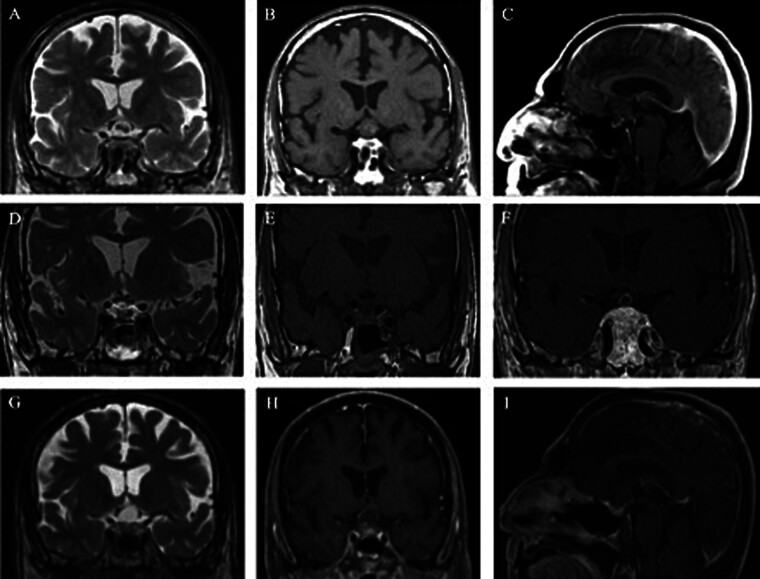
Preoperative coronal T2 and T1 ( **A, B** ), and sagittal ( **C** ) CT imaging. Postoperative coronal T1 and T2 ( **D, E** ), and T1 postcontrast ( **F** ) coronal CT. Coronal T1 and T2 ( **G, H** ), and postcontrast sagittal views of cyst recurrence.

This systematic review was prompted by a case of RCC recurrence at our institution with a histological diagnosis of a papillary CP after repeat surgery. The patient is a 74-year-old male with a history of Crohn's disease, hypogonadism, and osteoporosis who presented to our institution with progressive peripheral vision loss. MRI demonstrated a suprasellar cystic lesion with compression of the optic chiasm and extension into the third ventricle ( Fig. 2A–C ). Ophthalmologic evaluation identified decreased visual acuity, decreased color vision in both eyes, optic neuropathy in the left eye, and chiasmopathy in the right eye. Preoperative endocrine evaluation demonstrated panhypopituitarism with low testosterone, ACTH, and cortisol, for which he was started on replacement glucocorticoid therapy.

**Fig. 3:**
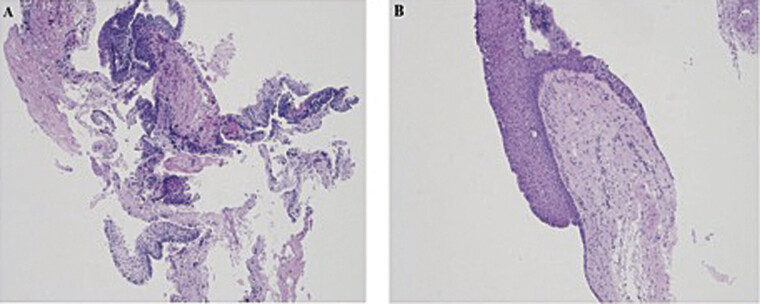
A 73-year-old male with vision loss found to have a suprasellar cystic lesion. ( **A** ) Neuropathology of the initial resection showed the cystic lesion with a pseudostratified squamous lining, felt to be most compatible with a Rathke's cleft cyst with squamous metaplasia. Immunohistochemical (IHC) stain for BRAF V600E was negative (not shown), and the attempted BRAF V600 qPCR assay resulted in a quantity not sufficient. An additional attempt to re-probe for BRAF V600E on a prior nonreactive immunostained slide was also negative. Image A. Hematoxylin and eosin (H&E)-stained slide, 40× total magnification. ( **B** ) Neuropathology at lesion recurrence approximately 6 months later again demonstrates fragments of pseudostratified squamous epithelium observed here with a discrete transition from attenuated Rathke's cleft epithelium of the pituitary pars intermedia. IHC stain for BRAF V600E was interpreted as equivocal due to exceedingly faint staining (not shown). The BRAF V600 qPCR assay was positive for an alteration in the BRAF V600 codon, in which the specific alteration, that is, specific BRAF V600E/E2/D, is not identified by this study. Repeat immunostaining for BRAF V600E showed similar results as the initial stain, interpreted as faint positive (not shown). Image B. Hematoxylin and eosin (H&E)-stained slide, 40× total magnification.

The patient underwent EETA for cyst drainage and partial cyst wall resection ( Fig. 2D–F ). Postoperatively, he developed permanent DDAVP deficiency, managed with replacement therapy. Pathologic findings demonstrated squamous epithelium with SM without wet keratin or calcifications and negative immunohistochemistry (IHC) staining for beta-catenin and BRAF V600E, consistent with a RCC with SM ( Fig. 3A ). Although visual function initially improved, the patient experienced visual decline again 4 months postoperatively. Repeat imaging revealed a recurrent suprasellar lesion, prompting repeat EETA ( Fig. 2G–I ). With the progressive nature of this patient's cyst, a more aggressive cyst wall resection was performed. Histopathologic evaluation again showed RCC with SM, but this time with positive IHC staining for BRAF p.V600E ( Fig. 2B ). Outside pathologic review confirmed the specimen as being a BRAF mutation-positive papillary CP, CNS WHO grade 1. Immunohistochemical results are summarized in Table 2 .

**Table 2:** Comparison of histopathological findings from the initial pathology after the first operation and the pathology from the second operation

	BRAF V600E	CK5/6	CK8/18	CK7	CK8	Ki67 (%)
Initial pathology	Negative	Strong diffuse	Strong diffuse	apical	Weak to moderate blush	<1
Second pathology	Positive	Strong diffuse	Strong diffuse	Strong apical	Moderate to strong apical	2–3

## Discussion

### Differentiation of RCC and CP

The relationship between RCCs and CPs has been a widely debated topic. 17 18 19 20 Similarities in imaging findings, histologic features, and clinical presentation create substantial diagnostic challenges. 21 Our case demonstrates this dilemma: an initial diagnosis of RCC was later revised to a PCP after repeat resection and more extensive tissue analysis. Our case illustrates the need for identifying distinguishing characteristics of RCCs and CPs to assist with initial diagnosis. We conducted a systematic review to discuss characteristics commonly used to distinguish RCCs and CPs.

### Histological Features

Histology remains the cornerstone of diagnosis, yet the relationship between RCCs and CPs can make this classification difficult. 22 RCCs classically present with ciliated cuboidal or columnar epithelium and occasionally SM. 23 By contrast, ACPs typically demonstrate features such as stratified squamous epithelium, peripheral palisading of nuclei, calcifications, wet keratin, and stellate reticulum, while PCPs are typically solid, not calcified, and appear homogenous. 23 24 Transition features, such as SM, blur these distinctions and contribute to misclassification. 22 The BRAF p.V600E is a well-known genetic hallmark of CPs and is routinely tested using VE1 IHC. However, RCCs with SM may yield similar staining, and in some cases, initially diagnosed as RCCs were later reclassified as CPs after confirmatory IHC revealed BRAF p.V600E mutation. 13 14 22 25 26 Schweizer et al reported three such cases where lesions originally classified as RCCs were later determined to be PCPs based on BRAF positivity and reassessment of the patient's clinical and radiographical features. They concluded that the presence of this mutation in RCCs shouldn't be thought of as a risk factor for recurrence, but rather the likelihood that the lesion is a PCP. 13 It has therefore been suggested to conduct confirmatory DNA sequencing following immunohistochemical analysis for BRAF p.V600E to fully differentiate RCCs and PCPs. 13 22 26 27

Not all cases are as straightforward. Alomari et al reported a pediatric case with overlapping RCC and CP histology and negative BRAF IHC, hypothesizing possible transformation. 8 In our case, the initial lesion tested negative for BRAF p.V600E, whereas recurrence was positive, suggesting sampling bias and underscoring the importance of comprehensive tissue analysis. Additional biomarkers such as P53 and Ki67 have shown prognostic value in CPs, with higher expression linked to a greater risk of recurrence. 28 However, their role in differentiating RCCs and CPs has not yet been defined. Future work exploring these markers in RCCs could clarify whether they can assist in ambiguous cases with transitional features.

### Imaging Features

To assist with preoperative diagnosis, imaging modalities have continued to be utilized to assist with distinguish RCCs and CPs. RCCs typically appear as nonenhancing cysts with homogenous T2 hypointensity and may contain intracystic nodules, reported in as many as 45% of cases. 29 30 This is in comparison to CPs, where the cyst wall enhances with contrast and T2 images are hyperintense. 29 Additionally, CPs often demonstrate rim enhancement, mixed cystic-solid morphology, and calcifications. 29 30 31 32 However, the overlap between RCCs and CPs is frequent. Although calcifications are typically seen in CPs, Raghunath et al reported that three out of their nine patients with RCCs presented with calcifications in their preoperative imaging, contributing to their original diagnosis of CP. 29

Novel techniques may improve diagnostic accuracy. Contrast-enhanced (CE) 3D T2-FLAIR MRI has shown promise in differentiating RCCs and CPs. In a study using CE 3D T2-FLAIR MRI to observe wall enhancement in RCCs and CPs, results showed wall enhancement in all CP participants and only one RCC participant. The authors concluded that CE 3D T2-FLAIR MR imaging may be a beneficial diagnostic tool for distinguishing RCCs from cystic CPs. 31 In another study, Yang et al described distinct imaging patterns which displayed an upward/vertical growth pattern with superior optic chiasm displacement in RCCs and a backward pattern with ventral displacement of the optic chiasm in CPs. 32 Although these studies show promise for imaging modalities that differentiate RCCs and CPs, these findings require validation in larger cohorts.

The case we present reflects the challenges in preoperative imaging as a diagnostic modality. Initial imaging findings for our patient were nonspecific, leading to an initial diagnosis of RCC. These findings emphasize the complexity of diagnosing RCCs and CPs based on imaging alone and highlight the potential for developing more reliable diagnostic tools and criteria. 33 34 35 36 37 38 39 40

### Limitations

We acknowledge that our study has limitations. First, the retrospective nature of included studies and inconsistent use of confirmatory DNA sequencing limit the ability to determine whether transformation represents disease progression or diagnostic misclassification. Second, our single-institution case was supported by a small number of published reports, limiting the sample size and reducing the generalizability of our findings. Lastly, the rarity of reported cases may present selection and reporting bias. Despite these limitations, this review synthesizes the most comprehensive evidence to date on the potential transition and diagnostic challenge between RCCs and CPs.

## Conclusion

Distinguishing RCCs from CPs remains a major diagnostic challenge due to overlapping clinical, radiographic, and histologic features, particularly when SM is present. While BRAF p.V600E IHC serves as a useful adjunct, confirmatory sequencing is essential to avoid misclassification. In our case, the initial specimen lacked BRAF p.V600E, whereas the recurrent lesion was positive, suggesting sampling bias and underscoring the need for comprehensive tissue evaluation. Advanced imaging techniques such as CE 3D T2-FLAIR MRI show potential for improving preoperative differentiation but require further validation. Ultimately, accurate diagnosis relies on an integrated, multimodal approach combining clinical, imaging, and molecular findings to guide management and optimize outcomes.
